# Impact of delirium on postoperative frailty and long term cardiovascular events after cardiac surgery

**DOI:** 10.1371/journal.pone.0190359

**Published:** 2017-12-29

**Authors:** Masato Ogawa, Kazuhiro P. Izawa, Seimi Satomi-Kobayashi, Yasunori Tsuboi, Kodai Komaki, Yasuko Gotake, Yoshitada Sakai, Hiroshi Tanaka, Yutaka Okita

**Affiliations:** 1 Division of Rehabilitation Medicine, Kobe University Hospital, Kobe, Japan; 2 Department of International Health, Kobe University Graduate School of Health Sciences, Kobe, Japan; 3 Division of Cardiovascular Medicine, Department of Internal Medicine, Kobe University Graduate School of Medicine, Kobe, Japan; 4 Division of Cardiovascular Surgery, Department of Surgery, Kobe University Graduate School of Medicine, Kobe, Japan; 5 Division of Rehabilitation Medicine, Kobe University Graduate School of Medicine, Kobe, Japan; University of Milano, ITALY

## Abstract

**Background:**

Postoperative delirium (POD) is a common and critical complication after cardiac surgery. However, the relationship between POD and postoperative physical frailty and the effect of both on long-term clinical outcomes have not been fully explored.

**Objective:**

We aimed to examine the associations among POD, postoperative frailty, and major adverse cardiac events (MACE).

**Design:**

This was a prospective cohort study.

**Methods:**

We studied 329 consecutive patients undergoing elective cardiac surgery. The intensive care delirium screening checklist was used to assess POD. Postoperative frailty was defined by handgrip strength and walking speed. Patients were subsequently followed-up to detect MACE.

**Results:**

POD was present in 13.2%, while the incidence of postoperative frailty was 27.0%. POD was independently associated with development of postoperative frailty (adjusted odds ratio = 2.98). During follow-up, MACE occurred in 14.1% of all participants. On multivariate Cox proportional hazard analysis, POD (adjusted hazard ratio (HR) = 3.36), postoperative frailty (HR = 2.21), postoperative complications (HR = 1.54), and left ventricular ejection fraction (HR = 0.95) were independently associated with increased risk of MACE.

**Limitations:**

It is a single-center study with a risk of bias. We did not investigate follow up cognitive function.

**Conclusions:**

POD was a predictor of postoperative frailty after cardiac surgery. Both postoperative frailty and POD were associated with the incidence of MACE, while POD was the stronger predictor of MACE. Thus, POD and frailty play critical roles in the risk stratification of patients undergoing cardiac surgery.

## Introduction

Delirium is defined as a temporary state of mental confusion and fluctuating consciousness, and it occurs in as many as 30% to 50% of patients after cardiac surgery [1].

A previous study showed that postoperative delirium (POD) increased with aging and resulted in a high rate of postoperative complications [2]. It was previously believed that delirium was a reversible cognitive impairment; however, recently, long-term adverse effects due to delirium were revealed.

Gottesman et al. [3] reported that POD after cardiac surgery was a strong predictor of mortality up to 10 years after the surgery. Another study also demonstrated that POD was associated with a significant decline in cognitive ability during the first year after cardiac surgery and no improvement was observed regardless of age [4]. Furthermore, patients with persistent delirium have been shown to be less likely to regain normal activities of daily living [5]. Thus, delirium can cause physical, mental, and social vulnerability in patients.

In contrast, many studies have described the relationship between frailty and cardiovascular disease [6–8]. Two studies recently showed that frailty in itself was a marker for adverse outcomes in patients with cardiovascular diseases [9, 10]. The exact cause of why frailty is related to adverse outcomes remains unknown. However, it may be attributable to the systemic effects of cardiovascular disease, such as activation of inflammatory and neurohumoral processes and limitation of physical activity[11]. Such mechanisms may lead to worse clinical outcomes.

Nevertheless, little evidence is available on the relationship between POD, postoperative frailty, and cardiovascular events. Elderly people with diminished preoperative physiologic reserves could be at a high risk of further reduction in their reserve capacity due to delirium. Thus, our hypothesis was that POD is independently associated with postoperative frailty after adjusting for other clinical characteristics and is a marker for subsequent clinical events. Therefore, we aimed to examine the associations among postoperative delirium, postoperative frailty, and long-term cardiovascular events.

## Materials and methods

### Study population

Between September 2011 to December 2015, 368 consecutive patients who were admitted to Kobe University Hospital were screened. We enrolled a cohort of inpatients in whom cardiopulmonary bypass was used in procedures, such as coronary artery bypass surgery (CABG), valve replacement or repair, or CABG with concomitant valve replacement or repair. Exclusion criteria included patients who underwent off-pump CABG; had neurological, peripheral vascular, orthopedic, or pulmonary disease; or had an incomplete assessment of delirium. All patients received postoperative rehabilitation starting the day after the surgery, according to the Japanese Circulation Society guidelines for rehabilitation in patients with cardiovascular disease [12]. The present study complied with the principles of the Declaration of Helsinki regarding investigations in human subjects and was approved by the Kobe University Institutional Review Board. Written informed consent was obtained from each patient before surgery.

### Clinical characteristics of the patients

Medical records were reviewed to obtain information about demographic characteristics. Baseline characteristics evaluated included age, sex, body mass index, left ventricular ejection fraction (LVEF), estimated glomerular filtration rate (eGFR), brain natriuretic peptide (BNP) levels, serum hemoglobin levels, serum albumin levels, comorbidities, medications, and operative risk scores such as the European System for Cardiac Operative Risk Evaluation (EuroSCORE) II [13]. Laboratory data and cardiac echocardiography were evaluated within 1 week before cardiac surgery and just before discharge. Operative clinical variables recorded included the type of cardiac operation (CABG, cardiac valve replacement, or combined CABG and valve replacement), the duration of cardiac surgery, cardiopulmonary bypass time, and aortic cross-clamp time in minutes.

Postoperative clinical variables recorded included hospital mortality, length of the intensive care unit (ICU) stay, and postoperative complications associated with surgery. We classified postoperative complications as follows: death (secondary to any major cardiac event); postoperative myocardial infarction; stroke; spinal cord injury; postoperative renal failure, defined as a new requirement for hemodialysis; cardiogenic syncope or serious ventricular dysrhythmias such as ventricular tachycardia that required defibrillation; septic shock; cardiac tamponade; hemorrhage requiring reoperation; and deep wound infection that affected the sternum, muscles, and/or mediastinum and required antibiotic treatment and/or surgical debridement.

The primary end points for this study were major adverse cardiac events (MACE) after discharge, which included cardiovascular death, non-fatal myocardial infarction (MI), hospital readmission for worsening heart failure or angina, and stroke. The patients were followed-up as outpatients, and the date and cause of any reported event determined during regularly scheduled outpatient visits was confirmed by review of hospital medical records.

### Delirium assessment

Delirium assessments were conducted every 8 hours by trained bedside nurses starting the first day after surgery up until 5 days after surgery using the Intensive Care Delirium Screening Checklist (ICDSC) [14]. The assessment of delirium was also checked retrospectively by research staff using the patients’ medical records. The reliability and validity of the ICDSC for the detection of delirium have already been proven [14]. The ICDSC score ranges from 0 to 8, with a score of 4 or higher indicating clinical delirium. Patients with a diagnostic ICDSC score of ≥4 at any assessment point were considered likely to have delirium.

### Measurement of frailty

In this study, we assessed frailty by measuring handgrip strength and usual walking speed. Handgrip strength was measured with a grip strength dynamometer (T.K.K.5401; Takei Scientific Instruments Co., Ltd., Niigata, Japan) [15]. Three measurements were made of each hand, while watching for a possible Valsalva effect. We used the highest value of the right or left handgrip strength according to the standard protocol [16]. Walking speed was measured twice at usual speed as described elsewhere [17]. A 4-m section of the walkway was marked off by two lines, and space and time were allowed for acceleration and deceleration. Participants were allowed to use canes, but not assistance by a caregiver. Frailty was defined as diminished handgrip strength (<26 kg for men and <18 kg for women) and/ or usual walking speed (<0.8 m/s) as described elsewhere and according to the definition of declining physical performance by the Asian working group for sarcopenia [18]. We assessed preoperative frailty within one week before surgery and postoperative frailty just before hospital discharge.

### Statistical analysis

We conducted statistical analyses after confirming that the data were normally distributed using the Shapiro-Wilk test. We compared clinical characteristics based on the presence or absence of MACE or postoperative frailty, and with or without POD using an independent *t*-test or chi-square test. To analyze factors affecting postoperative frailty and POD, a logistic regression analysis was used to examine each association between the incidence of frailty or POD and each variable. In this analysis, the incidence of frailty or POD was used as the dependent variable, while the clinical characteristics were independent variables. Factors in univariate analysis showing *P* values <0.10 were entered simultaneously into a multivariate logistic regression model. To analyze factors affecting MACE, multivariate analysis was performed using the Cox proportional-hazards regression model to simultaneously adjust for potential confounding variables. In this analysis, the incidence of MACE was used as the dependent variable, while independent variables included clinical characteristics, POD, and frailty. Subsequently, Kaplan-Meier survival statistics were used to examine the time to a first event, and a log-rank analysis was applied to compare event-free survival according to the incidence of POD and frailty. All statistical analyses were performed using R (The R Foundation for Statistical Computing, Vienna, Austria). The statistical significance level was set at *p* <0.05.

## Results

### Patient characteristics

**Fig 1:**
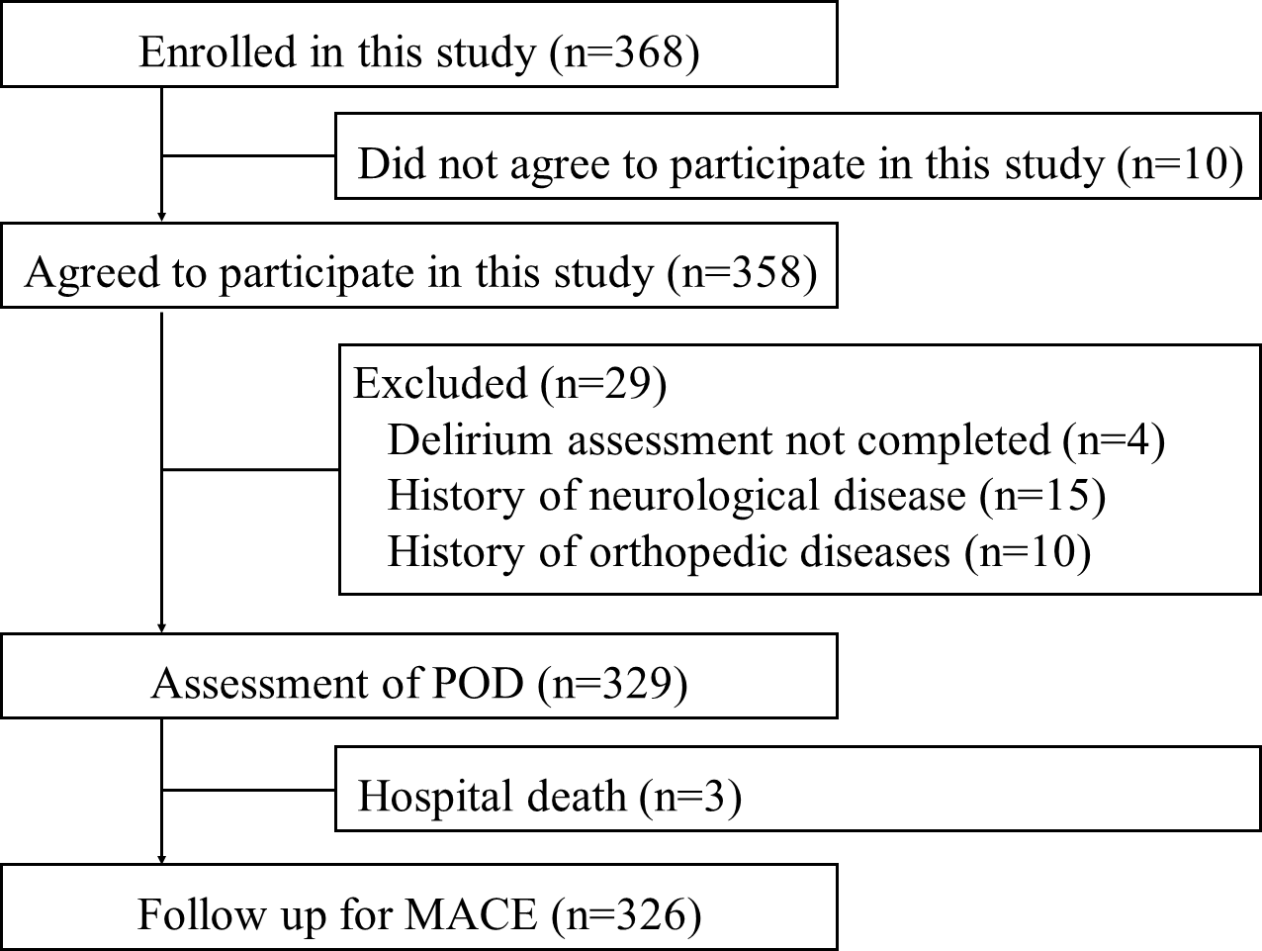
The flow chart displays the patient flow during the study. POD, postoperative delirium; MACE, major adverse cardiac events.

Of the 368 patients, 42 were excluded from the study based on the inclusion and exclusion criteria. Of the 42 excluded patients, 10 refused to participate in this study, 4 did not complete the POD assessment, 15 had neurological disease, 10 had orthopedic issues, and 3 died in the hospital. Thus, 326 patients were included in the study (Fig 1). As shown in Table 1, the mean age of the patients was 68.6±14.8 years and 44.2% were female. Comorbidities present were hypertension (52.5%), diabetes mellitus (22.4%), dyslipidemia (28.8%), and atrial fibrillation (18.4%). The mean operation time was 354.3±138.3 min, and the incidence of postoperative complications was 12.3%. The median follow-up period was 311 days (interquartile range, 132–560).

**Table 1:** Comparison of baseline characteristics in patients with or without MACE, and with or without postoperative frailty.

	Total	MACE (+)	MACE (-)	*t* or χ2 value	*p* value	Postoperative frailty (+)	Postoperative frailty (-)	t or χ2 value	*p* value
Number	326	46	280			88	238		
Age, years	68.6 ± 14.8	69.2 ± 15.4	68.1 ± 14.8	0.47	.64	77.5 ± 8.9	65.4 ± 15.3	6.71	< .0001
Female, n (%)	144 (44.2)	21 (45.7)	123 (43.9)	1.70	.19	54 (61.4)	90 (37.8)	22.72	< .0001
Body mass index, kg/m	23.1 ± 3.9	23.3 ± 4.3	23.0 ± 3.9	0.30	.76	22.3 ± 3.7	23.3 ± 4.0	-1.65	.11
Comorbidities									
Atrial fibrillation	60 (18.4)	11 (23.9)	49 (17.5)	1.66	.20	12 (13.6)	48 (20.2)	1.60	.19
Hypertension	171(52.5)	30 (65.2)	141 (50.4)	4.34	.03	48 (54.5)	123 (51.7)	2.33	.12
Dyslipidemia	94 (28.8)	18 (39.1)	76 (27.1)	3.62	.05	25 (26.1)	69 (29.0)	0.01	.98
Diabetes mellitus	73 (22.4)	15 (32.6)	58 (20.7)	3.62	.06	30 (34.1)	43 (18.1)	12.71	.0004
Euroscore II	6.3 ± 2.8	6.9 ± 3.4	6.2 ± 2.6	1.28	.20	6.9 ± 3.1	6.1 ± 2.5	1.52	.15
Types of surgery, n (%)									
Valve	206 (63.2)	25 (54.3)	181 (64.6)	1.80	.35	49 (55.7)	157 (66.0)	3.74	.15
Isolated CABG	29 (8.9)	5 (10.9)	24 (8.6)	―	―	9 (10.2)	20 (8.4)	―	―
Valve + CABG	91 (27.9)	16 (34.8)	75 (26.8)	―	―	30 (34.1)	57 (23.9)	―	―
Duration of surgery, min	354.3 ± 138.3	380.4 ± 126.4	348.1 ± 122.6	1.56	.12	333.1 ± 112.2	361.8 ± 126.8	-1.58	.11
Preoperative frailty, n (%)	22 (6.7)	6 (13.0)	16 (5.7)	2.70	.10	21 (23.9)	1 (0.4)	48.09	< .0001
**Postoperative parameters**									
Ejection fraction, %	60.0 ± 12.8	51.3 ± 15.2	61.1 ± 11.8	-4.80	< .0001	60.0 ± 12.8	60.1 ± 12.6	-0.02	.98
Laboratory data									
BNP, pg/ml	224.5 ± 369.2	363.1 ± 527.8	204.2 ± 337.2	2.50	.01	376.5 ± 532.0	176.2 ± 284.0	4.19	< .0001
eGFR, ml/min/1.73 m^2^	62.5 ± 22.2	60.0 ± 22.8	62.7 ± 22.1	-0.73	.47	53.0 ± 19.8	65.9 ± 22.1	-4.55	< .0001
Hemoglobin, g/dl	10.3 ± 1.18	10.2 ± 1.18	10.3 ± 1.13	0.35	.72	10.3 ± 1.2	10.2 ± 1.2	0.63	.53
Albumin, g/dl	3.1 ± 0.4	3.06 ± 0.38	3.11 ± 0.40	-0.74	.46	3.0 ± 0.4	3.1 ± 0.4	-2.28	.04
Medication, n (%)									
β-blocker	182 (55.8)	26 (56.5)	156 (55.7)	0.11	.74	47 (53.4)	135 (56.7)	0.07	.79
ACE-I / ARB	95 (29.1)	14 (30.4)	81 (28.9)	0.03	.87	25 (28.4)	70 (29.4)	0.11	.74
CCB	69 (21.2)	13 (28.3)	56 (20.0)	1.83	.18	15 (17.0)	54 (22.7)	1.83	.18
Diuretics	127 (39.0)	23 (50.0)	104 (37.1)	3.39	.07	38 (43.2)	89 (37.4)	2.06	.15
Length of ICU stay, day	2.4 ± 3.0	3.0 ± 1.4	2.3 ± 2.0	1.83	.27	3.1 ± 3.7	2.1 ± 2.1	2.35	.02
POD, n (%)	43 (13.2)	13 (28.3)	30 (10.7)	15.7	< .0001	19 (23.8)	28 (12.0)	5.93	.001
Postoperative complication, n (%)	40 (12.3)	10 (21.7)	30 (10.7)	9.70	.001	13 (14.8)	27 (11.3)	0.39	.53
Postoperative frailty, n (%)	88 (27.0)	30 (65.2)	58 (20.7)	12.6	< .0001	―	―	―	―
Postoperative MACE, n (%)	46 (14.1)	―	―	―	―	22 (25.0)	24 (10.1)	10.60	.0013

MACE: major cardiac adverse event; BNP: brain natriuretic peptide; eGFR: estimated glomerular filtration rate; ACE-I: angiotensin converting enzyme inhibitor; ARB: angiotensin II receptor blocker; CCB: calcium channel blocker; CABG: coronary artery bypasses grafting; ICU: intensive care unit; POD: postoperative delirium

Data are expressed as mean ± standard deviation or number (percentage).

### Incidence of POD after surgery

In our cohort, POD occurred in 44 patients (13.4%) according to the ICDSC criteria (Table 2). Individuals with POD were much older than non-POD patients (POD vs. non-POD; 72.8 ± 12.6 vs. 67.8 ± 14.7 years) and had lower preoperative eGFR (50.7 ± 20.5 vs. 60.7 ± 21.6 ml/min/1.73 m^2^; *p* <0.05 for each). Patients with POD stayed significantly longer in the ICU at a mean of 4.3 ± 5.2 days compared with non-POD patients (2.0 ± 1.5 days; *p* <0.0001). The prevalence of preoperative frailty was 18.6% in the patients with POD, while 4.9% in the patients with non-POD (*p* = 0.003). On the other hand, preoperative BNP and LVEF, comorbidities, and type of surgery were not significantly different between the two groups. In multivariate analysis of risk factors for the development of POD, length of ICU stay and preoperative frailty remained statistically significant after adjustment for other confounding factors (*p* <0.05 for each) (Table 3)

**Table 2:** Comparison of preoperative and intraoperative risk factors for delirium.

	Total	Delirium (+)	Delirium (-)	*t* or χ2 value	*p* value
Number	326	43 (13.2)	283 (86.8)		
Age, years	68.6 ± 14.8	72.8 ± 12.6	67.8 ± 14.7	2.18	.03
Female, n (%)	144 (44.2)	18 (41.9)	126 (44.5)	0.03	.85
Body mass index, kg/m	24.2 ± 3.9	24.2 ± 3.7	24.1 ± 4.0	0.07	.95
Comorbidities					
Atrial fibrillation	60 (18.4)	13 (30.2)	47(16.6)	2.73	.11
Hypertension	171(52.5)	27 (62.8)	144 (50.9)	0.60	.44
Dyslipidemia	94 (28.8)	14 (32.6)	80 (28.3)	0.02	.88
Diabetes mellitus	73 (22.4)	8 (18.6)	65 (23.0)	0.75	.39
Euroscore II	6.3 ± 2.8	6.6 ± 2.7	6.3 ± 2.8	0.73	.47
Ejection fraction, %	62.8 ± 10.8	59.9 ± 13.7	63.4 ± 12.4	-1.33	.18
Laboratory data					
BNP, pg/ml	171.2 ± 220.8	212.8 ± 199.9	165.5 ± 270.9	0.79	.43
eGFR, ml/min/1.73 m^2^	59.2 ± 21.7	50.7 ± 20.5	60.7 ± 21.6	-2.89	.004
Hemoglobin, g/dl	12.5 ± 1.9	12.1 ± 1.8	12.6 ± 2.0	-1.37	.17
Albumin, g/dl	3.9 ± 0.5	3.9 ± 0.5	4.0 ± 0.5	-1.08	.28
Medication, n (%)					
β-blocker	134 (41.1)	20 (46.5)	114 (40.3)	0.37	.54
ACE-I / ARB	155 (47.5)	25 (58.1)	130 (45.9)	2.18	.14
CCB	105 (32.2)	12 (27.9)	93 (32.9)	0.22	.64
Diuretics	129 (39.6)	19 (44.2)	110 (38.9)	0.25	.62
Preoperative frailty, n (%)	22 (6.7)	8 (18.6)	14 (4.9)	9.00	.003
Types of surgery, n (%)					
Valve	206 (63.2)	29 (67.4)	177 (62.5)	0.54	.77
Isolated CABG	29 (8.9)	4 (9.3)	25 (8.8)		
Valve + CABG	91 (27.9)	10 (23.3)	81 (28.6)		
Duration of surgery, min	354.3 ± 138.3	390.7 ± 138.3	344.8 ± 112.5	2.4	.02
Duration of CPB, min	179.5 ± 75.2	191.1 ± 77.1	175.7 ± 69.3	1.28	.20
Aortic cross-clamp time, min	108.9 ± 68.3	114.3 ± 64.4	98.7 ± 68.0	1.99	.05
Length of ICU stay, day	2.4 ± 3.0	4.3 ± 5.2	2.0 ± 1.5	4.91	< .0001

BNP: brain natriuretic peptide; eGFR: estimated glomerular filtration rate; ACE-I: angiotensin converting enzyme inhibitor; ARB: angiotensin II receptor blocker; CCB: calcium channel blocker; CABG: coronary artery bypasses grafting; CPB: cardiopulmonary bypass; ICU: intensive care unit

Data are expressed as mean ± standard deviation or number (percentage).

**Table 3:** Univariate and multivariate analysis of risk factors for development of postoperative delirium.

Variables	Univariate model		Multivariate model	
	OR (95% CI)	*p* value	OR (95% CI)	*p* value
Age	1.02 (1.01–1.41)	.02	1.01 (0.98–1.12)	.22
Sex, female	0.94 (0.50–1.78)	.85		
eGFR	0.98 (0.95–0.99)	.004	0.98 (0.97–1.02)	.18
Duration of surgery	1.01 (1.00–1.02)	.02	1.00 (0.99–1.00)	.28
ICU stay	1.28 (1.06–3.10)	.001	1.15 (1.04–1.97)	.02
Preoperative frailty	4.00 (2.04–4.35)	< .0001	2.85 (1.19–4.26)	.03

eGFR: estimated glomerular filtration rate; ICU: intensive care unit; OR, odds ratio; CI, confidence interval.

### Postoperative frailty and its predictors

As shown Table 1, the incidence of postoperative frailty was 27.0% (88 of 326 patients). Patients with POD had an approximately twice increased risk of postoperative frailty (23.8% vs. 12.0%; *p* <0.0001). In multivariate analysis, POD [adjusted odds ratio (OR) = 2.98; 95% confidence interval (CI): 1.46–6.20], age (adjusted OR = 1.09; 95% CI: 1.03–1.29), BNP level (adjusted OR = 1.00; 95% CI: 1.00–1.03), and preoperative frailty (adjusted OR = 3.70; 95% CI: 1.26–12.10) were independently associated with developing postoperative frailty after adjusting for potential confounders (Table 4).

**Table 4:** Univariate and multivariate analysis of risk factors for postoperative frailty.

Variables	Univariate model		Multivariate model	
	OR (95% CI)	*p* value	OR (95% CI)	*p* value
Age	1.10 (1.03–1.39)	< .0001	1.09 (1.03–1.29)	< .0001
Sex, female	2.21 (1.00–4.79)	.04	2.98 (1.75–6.74)	.02
BMI	0.93 (0.79–1.04)	.16	―	―
Diabetes mellitus	2.62 (1.46–4.70)	.001	2.04 (0.79–5.20)	.14
eGFR	0.97 (0.60–0.99)	< .0001	0.97 (0.94–1.01)	.07
Albumin	0.68 (0.21–1.14)	.13	―	―
BNP	1.00 (1.00–1.02)	.004	1.00 (1.00–1.03)	.02
ICU stay	1.13 (0.98–1.99)	.10	―	―
POD	3.73 (2.13–6.68)	< .0001	2.98 (1.46–6.20)	.002
Preoperative frailty	15.75 (6.37–44.84)	.16	3.70 (1.26-12-10)	.02

eGFR: estimated glomerular filtration rate; BMI: body mass index; BNP: brain natriuretic peptide; ICU: intensive care unit; POD: postoperative delirium; OR, odds ratio; CI, confidence interval.

### Results for MACE

In this study, MACE after discharge occurred in 14.1% (46 of 326) of patients during follow up. The MACE were 4 events of MI, 29 events of heart failure, 8 events of stroke, and 5 events of cardiovascular death. Univariate associations with MACE after cardiac surgery are shown in Table 1. The presence of hypertension (*p* = 0.03), low preoperative cardiac function (*p* <0.0001), high BNP levels (*p* = 0.01), and postoperative complications (*p* = 0.001) were significantly associated with the development of MACE, while age, the use of cardioprotective medications, and types of surgery were not associated with the incidence of MACE. POD was significantly associated with a high incidence of MACE (28.3% vs. 10.7%; *p* <0.0001). Prevalence of postoperative frailty was 65.2% in the MACE group and 20.7% in the non-MACE group, indicating that the likelihood of MACE in frail patients was 3 times higher compared with that in the non-frail patients (*p* <0.0001), while preoperative frailty was not statistically significant for predicting MACE. The multivariate Cox proportional hazard model identified POD [adjusted hazard ratio (HR) = 3.36; 95% CI: 1.32–7.32], postoperative frailty (adjusted HR = 2.21; 95% CI: 1.01–4.82), postoperative complications (adjusted HR = 1.54; 95% CI: 0.64–3.43), and postoperative LVEF (adjusted HR = 0.95; 95% CI: 0.93–0.98) as independent predictors of MACE (Table 5).

**Table 5:** Independent predictors of MACE for patients undergoing cardiac surgery (Cox-hazard analysis).

Variables	Univariate model		Multivariate model	
	Hazard ratio (95% CI)	*p* value	Hazard ratio (95% CI)	*p* value
Age	1.01 (0.98–1.03)	.72	―	―
Sex, female	1.51 (0.82–2.80)	.19	―	―
POD	4.70 (2.31–9.12)	< .0001	3.36 (1.32–7.82)	.016
Postoperative frailty	2.39 (1.20–4.78)	.01	2.21 (1.01–4.82)	.047
Hypertension	1.61 (0.85–3.17)	.14	―	―
Diabetes mellitus	1.61 (0.82–3.02)	.16	―	―
Dyslipidemia	1.43 (0.76–2.64)	.26	―	―
BNP	1.01 (1.00–1.01)	.02	1.00 (1.00–1.00)	.62
Postoperative complication	2.45 (1.19–4.76)	.02	1.54 (0.64–3.43)	.01
LVEF	0.95 (0.93–0.97)	< .0001	0.95 (0.93–0.98)	.0003
Diuretics	1.66 (0.91–3.05)	.09	1.22 (0.56–2.69)	.62

POD: postoperative delirium; BNP: brain natriuretic peptide; LVEF: left ventricular ejection fraction.

**Fig 2:**
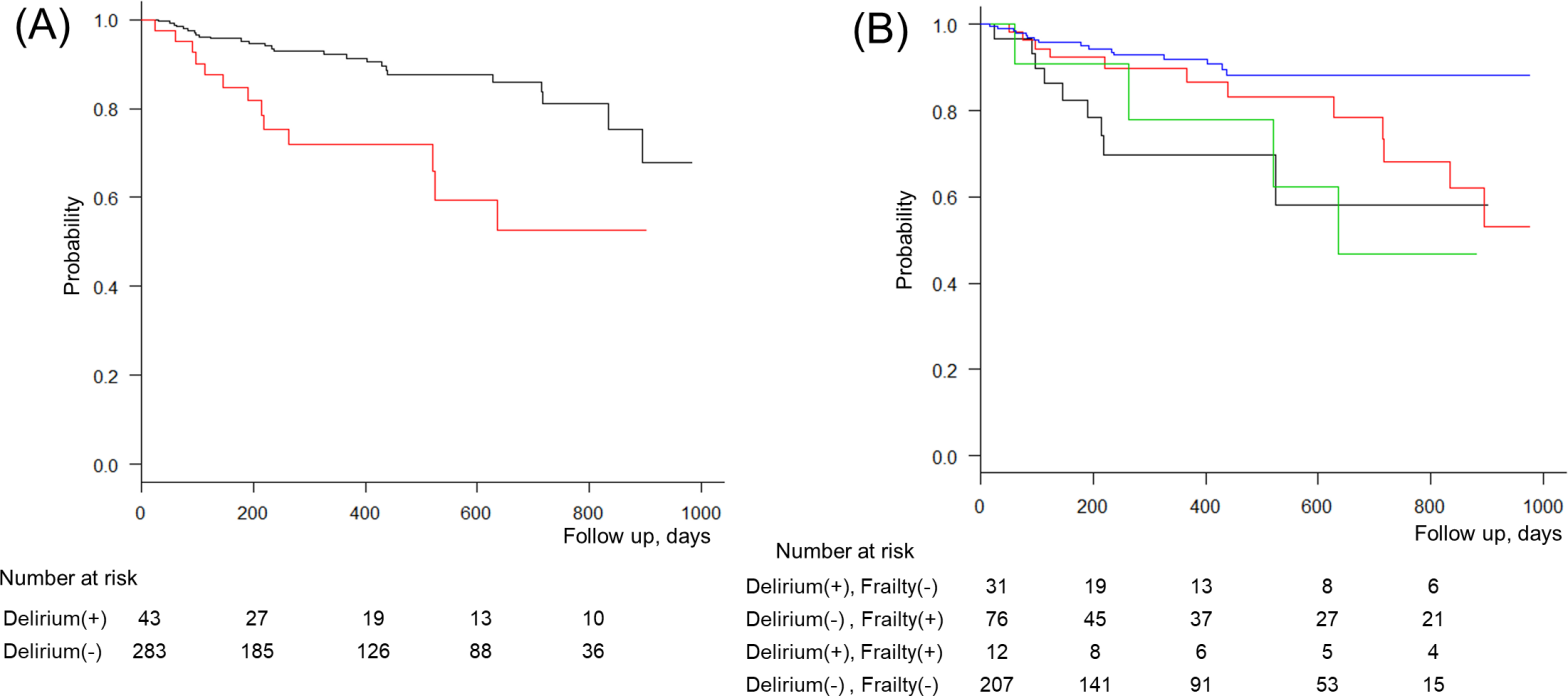
Kaplan–Meier curves for cumulative major adverse cardiac events for (A) presence (red line) or absence (black line) of postoperative delirium (Log-rank test, p = 0.000733), (B) a comparison of the four groups robust (blue line), delirium (black line), postoperative frailty (red line), and combination of both (green line) (Log-rank test, p = 0.00369).

The event-free survival curves of the four groups stratified by POD and postoperative frailty are shown in Fig 2. The Kaplan Meier analysis showed that POD and frailty were significantly associated with the incidence of MACE (Log-rank test *p* = 0.0036).

## Discussion

To the best of our knowledge, this is the first study to demonstrate the relationship between POD, postoperative frailty, and cardiovascular events in patients undergoing cardiac surgery. We found that both POD and postoperative frailty were independently associated with a worse clinical prognosis after adjustment for confounding variables.

It was reported that the incidence of POD after cardiac surgery varied with the type of cardiac surgery and ranged from 10% to 80% [19]. In our cohort, the incidence of POD was 14.1%, which is lower than that of other studies [20]. A possible explanation is that our study included only cases of elective surgery and excluded emergency or urgent surgery in patients at a high risk of POD [20]. Furthermore, the mean age of our cohort was lower (64.9 ± 16.2 years) than that of previous studies [21]. Systematic review has shown that increasing age is the most important risk factor for POD [22]. Taken together, it is conceivable that our cohort comprised patients with a relatively low risk of delirium.

The pathophysiology of delirium has not been fully clarified; however, neuroinflammation and endothelial dysfunction have been identified as factors leading to an increased risk of delirium [23]. We showed that duration of surgery was significantly longer in the patients with POD. Our result is in line with a previous study that demonstrated that POD was independently associated with longer surgery duration [24]. Prolonged duration of surgery led to longer cardiopulmonary bypass (CPB) times and aortic cross-clamp time. During cardiac surgery using cardiopulmonary bypass, neuroinflammatory damage of the brain may occur due to microembolism, reduced cerebral flow, and fluid shift. These neurotransmitters may be responsible for the occurrence of POD in our cohort.

In contrast, it is well known that cognitive impairment after delirium is very common and persists for at least 1 year [4]. O’Donnell et al. [25] showed that cognitive dysfunction was associated with an increased risk of cardiovascular events, which was similar to that in patients with a previous stroke. Furthermore, decline of cognitive function causes several unfavorable conditions, such as poor compliance with medication, daily self-weighing, and symptom monitoring; increasing depression symptoms; and decline of social activity and physical function [26–28]. These findings would explain why POD was strongly associated with increased MACE in the present study. Moreover, heart failure was the most frequent cause of MACE. Presuming that the difficulty of self-management may worsen heart failure, it is reasonable that POD was significantly associated with increased MACE. Furthermore, patients with POD were older, more likely to have a lower serum eGFR level, and more physically frail than those without POD. These background factors that cause POD due to a decreased total reserve capacity and vulnerability also affect the occurrence of MACE, not only POD itself.

Postoperative frailty was also an independent predictor of MACE in this study. Recently, several studies have investigated the relationship between frailty and worse outcomes such as falls and fractures, hospitalization, low quality of life, and high mortality in the general elderly population and in patients with heart failure [10, 29, 30]. Nevertheless, this is the first report to investigate frailty after cardiac surgery and its impact on prognosis. Frailty is considered to be a result of reduced physiological capacity [31]; it is caused by multiple factors, such as malnutrition, physical activity limitation, and cachexia, and can lead to unmanageable dysregulation of homeostasis. Thus, it is not surprising that the patients with frailty had negative health outcomes, and our results are in line with previous studies that demonstrated frailty in patients with heart failure [10]. On the contrary, preoperative frailty was not a predictor of MACE after discharge. It was thought that declining physical function due to delirium is more important and implicates the onset of MACE after discharge. Postoperative frailty caused decreased ability to mobilize and ambulate, and diminished physiological reserve and capacity to maintain homeostasis [32]. We think that these mechanisms partly explain the occurrence of MACE. In contrast, we had previously reported that low preoperative physical function was an independent predictor of POD after cardiac surgery [33]. Thus, delirium is thought to be both a cause and consequence of frailty.

It was also notable that POD was a stronger predictor of MACE than postoperative frailty. A previous study showed that cognitive impairment due to poor self-care management, defined as an inadequate decision-making process, was common in heart failure and may cause MACE [34]. Poor self-care management and confidence can be conducive to further functional limitation and frailty. Our findings underscore that intervention for prevention or treatment of POD is more important for frail patients.

In multivariate analysis, low LVEF and major postoperative complications were also identified as independent predictors of MACE. Uthamalingam et al. [35] reported the relationship between acute delirium and hospital readmissions with acute decompensated heart failure. In accordance with our results, they demonstrated that low LVEF and delirium were independent predictors of readmission. It is well known that patients with severe cardiac dysfunction have a higher risk of death [36]. In addition, it is interesting to note that Georgiadis et al.[37] have demonstrated that a decline in LVEF is related to partially diminished cerebrovascular reactive dilatory capacity in patients with chronic heart failure. We did not investigate brain metabolism in this study; however, a decline in cerebrovascular reserve capacity due to attenuated cardiac function could be responsible for POD and cognitive dysfunction after cardiac surgery. The effects of these mechanisms can be observed in part by the significant increase in the incidence of MACE in patients with low LVEF.

Various treatments for delirium have been suggested, including antipsychotic agents, almost none of which have been effective [19]. Thus, we should focus on the prevention of delirium rather than its treatment, especially because Vidán et al. [38] demonstrated that a multicomponent non-pharmacological intervention for preventing delirium was effective and improved quality of care. In their study, the interventions to prevent delirium, included education, orientation, sleep preservation, nutritional support, and early mobilization. It is our firm belief that, particularly among such multicomponent interventions, early mobilization is important and essential to prevent delirium and functional decline especially for older patients or patients with renal failure. Furthermore, because the long-term care of patients who develop POD is often overlooked, there is a need for long-term follow-up and education of caregivers to aid in the self-management of heart failure. Further studies are required to confirm the effectiveness of such early intervention.

### Study limitations

There are some limitations to this study. It is a single-center study with a risk of bias. We could not analyze age- or sex-related differences because of our small sample size. We investigated baseline cognitive function from all patients, but did not reassess them during follow up. The patients with POD had persistent cognitive impairment [4], although the details of cognitive deficit were unclear. We also used ICDSC to detect POD, whose validity and reliability in the detection of delirium has already been proven. Nevertheless, it was reported that the specificity of the ICDSC is lower than that of other screening tools such as the Confusion-Assessment-Method for the ICU[14]. Therefore, the incidence of POD might have been underestimated in our cohort.

## Conclusion

POD was an independent predictor of the incidence of postoperative frailty after cardiac surgery. Both postoperative frailty and POD were strongly associated with MACE at 1 year after surgery, while POD was the stronger predictor of MACE than frailty. Thus, POD and frailty play extremely important roles in the risk stratification of patients undergoing cardiac surgery. A multicomponent intervention to prevent delirium and frailty is a matter of considerable urgency.
